# Predictors of personality disorders in prisoners

**DOI:** 10.25122/jml-2021-0317

**Published:** 2022-04

**Authors:** Fayegh Yousefi, Mansor Abu Talib

**Affiliations:** 1. Department of Psychiatry, Faculty of Medicine, Kurdistan University of Medical Sciences, Sanandaj, Iran; 2. Social Determinants of Health Research Center, Kurdistan University of Medical Sciences, Sanandaj, Iran; 3. Department of Psychology, Faculty of Social Sciences & Liberal Arts, UCSI University, Kuala Lumpur, Malaysia

**Keywords:** personality disorder, risk factors, prisoners

## Abstract

Personality disorders can lead to difficult social or occupational functional processes rooted in chronic maladaptive thoughts, feelings, and behaviors. This study aimed to investigate factors of personality disorder in prisoners from the central prison of Sanandaj, Iran. We conducted a cross-sectional study, which included all prisoners in the central prison of Sanandaj, Iran. The study sample includes 343 prisoners, of which 329 were male, and 14 were female, selected by randomized multistage sampling method. Participants filled in Millon's multi-axis clinical questionnaire. The data were analyzed using Chi-square, multiple logistic regression, and bootstrap analysis. There were 183 participants without personality disorders (53.4%) and 99 participants (28.9%) with cluster B personality disorders (narcissistic, histrionic, anti-social, and borderline). Male gender (OR=0.07) and elementary education level (OR=0.18) have a significant relationship with cluster A personality disorders (paranoid, schizoid, schizotypal). Cluster B has a significant relationship only with the elementary education level (OR=0.27). Cluster C (avoidant, dependent, obsessive-compulsive personality disorder) has a significant relationship with male gender (OR=0.20), elementary education level (OR=0.30), unemployment (OR=2.64), theft crime types (OR=0.38) and disputes and assaults (OR=0.18). Based on these results, psychological and psychiatric interventions in prisoners are suggested.

## Introduction

Personality Disorders (PDs) can substantially lead to difficult social or occupational functioning processes induced by chronic maladaptive thoughts, feelings, and behaviors. These are prevalent in nearly 6% of the world's population [1–4]. The risks of violent behaviors seem to be identifiable via the significant role of a personality profile, including PDs and clinically significant traits of personality [2, 5].

Since the beginning of the 19^th^ century, mentally ill people have been widely identified to be more frequently engaged in violent crimes compared to healthy populations [6, 7]. Research has well documented a robust relationship of the different types of personalities, such as schizoid position, anti-social behavior, borderline disorder, and psychopathic attitude with violent crimes [5, 8]. Violence and mental disorder are linked with paranoid cognitive personality and narcissism (threatened egotism) [2]. This association for psychopathic people can be justified by such traits as pathological egocentricity, lack of empathy and remorse, shallow emotions, and low-threshold aggression [9]. 

Physically and psychologically inducing conditions for crime may be reinforced in prisons where living conditions are limited [10]. Eysenck believed that various crime types can arise from a combination of neurological factors and environmental circumstances. Moreover, he maintains that personality plays a main role in crime and is thus a decisive factor in criminal behaviors [11]. This hypothesis implies that some individuals have greater propensities for crime commitment [12]. Criminal acts are highly complicated due to being intertwined with medical, psychological, sociological, legal, and political issues [13]. Prisoners may be highly involved in prevalent psychopathic processes leading to lifelong psychiatric hospitalization and homelessness.

Nevertheless, considerable differences (3–73%) in psychopathic prevalence rates among prisoners have been reported in different countries [14]. As documented in most relevant studies, prisoner populations are most frequently involved in personality and substance abuse disorders [8]. Also, anti-social disorder has been mentioned by Zemestani *et al.* as the most frequent personality disorder among criminals [15]. For example, an investigation conducted on 228 prisoners in Kerman, Iran, indicated the involvements of 83.3% and 87.3% of the studied men and women in some types of personality disorders at the time of crime commitment, respectively. Major depressive disorder (MDD), dependent personality disorder (DPD), borderline personality disorder (BPD), and anti-social personality disorder (ASPD) represented the highest mental disorder rates among the mentioned population, respectively [16].

Several studies suggested the present applicability of certain male-related risk factors to females and the inability of violence committed by younger males and females [14, 17]. Prisoners usually come from socioeconomically deprived families with criminal behavior and mostly psychiatric disorder backgrounds [18]. Also, low and middle-income countries (LMICs) have accommodated the majority of the world's imprisoned populations [7]. The seven LMICs of Mexico, Brazil, Egypt in South Africa, Nigeria, Iran, India, and Malaysia comprise the only countries reported for mental disorder prevalence [10, 16, 19–21]. Some research has evidenced perpetrators at the subsequent risks of anti-social behaviors and entanglements in varied employment and relationship adjustments or other relevant problems [22].

The highest prevalence rates of personality disorders were related to theft (64.1%), drug addiction (60.9%), iniquity and murder (55.6%), drug trafficking (55.0%), and financial crimes (40.9%) based on crime type, respectively [16].

This study investigated personality disorders among prisoners in Sanandaj in Kurdistan Province, Iran. Our research on the prevalence rates of personality disorders in the studied prisoner population and their relations with criminal histories could add useful information to the existing literature.

The population under study was excluded from mental health services both during the conviction and post-release periods of the prisoners as they had been highly involved in personality disorders and their associated co-morbidities of alcohol and drug dependencies [23]. Nevertheless, the results may improve prisoners' criminal behaviors by giving insights into personality disorders and their dysfunctional traits as potential risk factors to be considered by future clinicians and researchers.

## Material and Methods

This study was cross-sectional and included prisoners from the central prison of Kurdistan in Sanandaj, Iran. The samples were determined by the Cochran formula, and there were 384 participants with a mean age of 34.39±8.16 years. Given the prison sections, we used a stratified random sampling method, and participants were selected based on the population from each section. 

The inclusion criteria of this study include having a high school degree to understand the questions of the questionnaire and being a prisoner in a prison center in Sanandaj. The exclusion criteria was mental retardation recognized by a clinical psychologist.

### Instruments

#### The Millon Personality Disturbance Questionnaire (MCMI-II)

The Millon Personality Disturbance Questionnaire (MCMI-II) is based on the Biological, Psychological, and Social Millon's Thinking Society, which includes 22 scales and is categorized into four categories: personality clinical patterns, severe personality trauma, clinical syndrome, and severe syndrome. This questionnaire assesses antisocial, selfish, passive-aggressive, obsessive-compulsive, schizoid, avoidance, demographics, and self-destructive personality disorders [24]. Also, this questionnaire contains 175 short-term self-explanatory sentences with yes-no-yes responses [24]. The internal consistency of the MCMI-II scales was reported by Kuder–Richardson 20 from 0.81 (delusional disorder) to 0.95 (depression) with a mean of 0.09 for all scales [25]. According to the preliminary validation results of Millon's clinical multi-axis scale in Iran, the coefficient of inclination of the scale is 0.85 with Kuder- Richardson, and its retest coefficient is 0.86 [25]. 

#### Checklist

The checklist includes variables such as gender, age, education, occupation, marital status, number of children, a record of imprisonment, term of imprisonment, and type of crime.

### Procedure

After approval of the plan, coordination with the central prison of Sanandaj was necessary. Then, as the central prison has different parts, the stratified random sampling method was used to ensure that proportion of prisoners from all parts of the prison were randomly selected. Based on some studies [26, 27], the diagnosis of the personality disorders in participants was determined via Millon Personality Disturbance Questionnaire (MCMI-II).

### Data analysis method

Data were analyzed using the SPSS-20 package. Descriptive statistics were used for background characteristics. Also, the Chi-square test was used to assess the relationship between these variables with different clusters. Multinomial regression was used for analyzing the risk ratio adjusted with a 95% confidence interval of each factor in the presence of other variables. Finally, bootstrap for estimated parameter and nominal regression were used.

## Results

A total of 343 prisoners participated in this study, of which 329 (95.91%) were male, and 14 (4.09%) were female. The number of participants without personality disorders was 183 (53.3%), and persons with cluster A personality disorder was 34 (9.9%), cluster B included 99 participants (28.9%), and cluster C included 27 participants (7.9%) (Table 1).

**Table 1.** Demographic characteristics of the responders

**Variables**		**Frequency**	**Percentage**
**Cluster**	A	34	9.9%
	B	99	28.9%
	C	27	7.9%
	Normal	183	53.4%
**Gender**	Male	329	95.9%
	Female	14	4.1%
**Education level**	Elementary	184	53.9%
	Diploma	115	33.5%
	≥Bachelor's degree	43	12.5%
**Job status**	Employee	21	6.1%
	Unemployed	74	21.6%
	Self-employment	248	72.3%
**Criminal type**	Theft	110	32.1%
	Financial crime	49	14.3%
	Murder	39	11.4%
	Disputes and assaults	62	18.1%
	Other crimes	83	24.2%
**Valid**		343	100%
**Missing**		0	
**Total**		343	

Table 1 shows that 32.1% of the participants were imprisoned for theft, while financial crimes, murder, disputes and assaults, and other crimes were 14.3%, 11.4%, 18.1%, and 24.2, respectively.

Table 2 shows the classification ratio of personality disorders in each of the following: people with a history of crime less than 5 years are more prevalent in cluster B and they are less than 40 years old. Also, single people make up the majority of people in cluster B.

**Table 2.** The comparison of personality disorders (Cluster A, B, and C) in case and control groups.

**Variables**	**Paranoid, schizoid schizotypal **	**Narcissistic histrionic, borderline, antisocial**	**Avoidant, dependent, OCDP**	**Normal**	**Chi-Square test**	**df**	**p**
**Groups**	n (%)	n (%)	n (%)	n (%)	11	3	0.008
**Case (sustenance abuse) **	13 (15)	19 (22)	12 (14)	39 (46)			
**Control**	21 (8.1)	80 (30)	15 (5)	144 (55)			

There were significant differences between normal people and people with personality disorder in variables such as gender (p=0.05), level of education (p=0.05), occupational status (p=0.03), and type of crime (p=0.01). In this study, there was no significant relationship between the marital status, criminal history, number of children, and age in normal people and persons with personality disorders in prisoners (Table 3).

**Table 3.** Association between demographic factors and other variables in the cluster A, B, and C personality disorders.

**Variable**	**Cluster A**	**Cluster B**	**Cluster C**	**Normal**	**Chi-Square test**	**P Value**
**Gender**					7.46	0.05
Male	32	93	24	180		
Female	2	6	3	3		
**Education levels**					12.55	0.05
Elementary	18	41	13	113		
Diploma	11	41	9	54		
Bachelor's degree and higher	5	17	5	16		
**Marriage status**					1.04	0.79
Married	15	41	14	77		
Single	19	58	13	106		
**Job-status**					13.93	0.03
Employee	0	6	4	11		
Unemployed	5	25	10	34		
Self-employment	29	68	13	138		
**Number of children**					1.09	0.77
Have not	17	45	13	95		
Have	17	54	14	88		
**Criminal type**					26.00	0.01
Theft	14	24	7	65		
Financial crime	2	17	3	27		
Murder	0	18	3	18		
Disputes and assaults	5	21	2	34		
Other crimes	13	19	12	39		
**History of crime**					2.43	0.48
<5	24	82	22	147		
≥5	10	17	5	36		
**Age**					1.80	0.61
<40	28	70	20	136		
≥40	6	29	7	47		

Cluster A – Paranoid, schizoid, schizotypal; Cluster B – Narcissistic, histrionic, borderline, and antisocial; cluster C – Avoidant, dependent, Obsessive-Compulsive Personality Disorder.

The multivariate analysis of factors related to personality disorders, gender, education level, occupational status, and type of crime was introduced into the logistic regression model. This analysis showed that male gender (OR=0.07) and elementary education level (OR=0.18) have a significant relationship with cluster A personality disorders and. Furthermore, cluster B has a significant relationship only with elementary education level (OR=0.27), and cluster C has a significant relationship with male gender (OR=0.20), elementary education level (OR=0.30), unemployment (OR=2.64), theft crime types (OR=0.38) and disputes and assaults (OR=0.18) (Table 4).

**Table 4.** Multinomial logistic regression results and Odds Ratio (OR) of personality disorder in prisoner.

**Cluster**	**Variable**	**P-Value**	**OR**	**95% Confidence interval for Esp.(B)**	
				**Lower**	**Upper**
**Cluster A: Paranoid, schizoid, chizotypal**					
**Gender**	Male	0.02	0.07	0.0	0.69
	Female	0	0	0	0
**Education levels**	Elementary	0.01	0.18	0.05	0.68
	Diploma	0.07	0.29	0.07	1.10
	Bachelor's degree and higher	.	.	.	.
**Job status**	Employee	.	.	.	.
	Unemployed	0.23	0.49	0.15	1.55
	Self-employment	.	.	.	.
**Criminal type**	Theft	0.21	0.57	0.23	1.37
	Financial crime	.	.	.	.
	Murder	.	.	.	.
	Disputes and assaults	0.05	0.30	0.09	1.02
	Other crimes	.0.01	0.18.	0.05.	0.68.
**Cluster B: Narcissistic, histrionic, borderline, antisocial**					
**Gender**	Male	0.27	0.43	0.09	1.95
	Female	.	.	.	.
**Education levels**	Elementary	0.0	0.27	0.11	0.68
	Diploma	0.24	0.59	0.24	1.43
	Bachelor's degree and higher	.	.	.	.
**Job status**	Employee	0.24	0.50	0.15	1.62
	Unemployed	0.19	1.52	0.80	2.88
	Self-employment	.	.	.	.
**Criminal type**	Theft	0.39	0.72	0.34	1.51
	Financial crime	0.88	0.93	0.39	2.23
	Murder	0.17	1.85	0.76	4.51
	Disputes and assaults	0.89	1.05	0.47	2.35
	Other crimes	.	.	.	.
Cluster C: Avoidant, dependent, Obsessive Compulsive Personality Disorder					
**Gender**	Male	0.08	0.20	0.03	1.27
	Female	.	.	.	.
**Education levels**	Elementary	0.12	0.30	0.06	1.37
	Diploma	0.36	0.50	0.11	2.21
	Bachelor's degree and higher	.	.	.	.
**Job status**	Employee	0.70	1.37	0.26	7.02
	Unemployed	0.04	2.64	1.01	6.88
	Self-employment	.	.	.	.
**Criminal type**	Theft	0.07	0.38	0.13	1.11
	Financial crime	0.08	0.26	0.06	1.18
	Murder	0.27	0.44	0.10	1.91
	Disputes and assaults	0.04	0.18	0.03	0.95

Results and odds ratio (OR) of personality disorder in prisoners are presented in Table 4. The bootstrap for parameter estimates results (Table 5) confirmed the results from the multinomial logistic regression.

**Table 5.** Bootstrap for parameter estimates results and odds ratio (or) of personality disorders in prisoners.

**Cluster**	**Variable**	**B**	**Bias**	**Bootstrap**	**95% Confidence Interval**	
				**P-Value**	**Lower**	**Upper**
**Paranoid, schizoid, schizotypal**						
**Gender**	Male	-2.61	-0.17	0.04	-20.62	13.92
	female	0	0		0	0
**Education levels**	Elementary	-1.68	0.08	0.02	-3.20	0.03
	Diploma	-1.23	0.06	0.08	-2.95	0. 71
	Bachelor's degree and higher	0	0		0	0
**Job status**	Employee	-21.39	0.70		-36.83	-11.03
	Unemployed	-0.69	-0.43	0.27	-8.75	0.39
	Self-employment	0	0		0	0
**Criminal type**	Theft	-0.56	-0.06	0.17	-1.54	0.19
	Financial crime	-0.06	-0.09	0.85	-1.20	0.70
	Murder	0.61	-0.06	0.23	-0.38	1.90
	Disputes and assaults	0.05	-0.01	0.93	-1.00	0.94
	Other crimes	0	0		0	0
**Narcissistic, histrionic, borderline, antisocial**						
**Gender**	Male	-0.83	-0.70	0.19	-16.45	0.49
	female	0	0		0	0
**Education levels**	Elementary	-1.27	-0.16	0.02	-2.44	-0.56
	Diploma	-0.51	-0.10	0.24	-1.63	0.28
	Bachelor's degree and higher	0	0		0	0
**Job status**	Employee	-0.69	-0.16	0.30	-2.99	0.58
	Unemployed	0.42	0.05	0.17	-0.25	1.06
	Self-employment	0	0		0	0
**Criminal type**	Theft	-0.32	-0.08	0.42	-0.99	0.60
	Financial crime	-0.06	-0.09	0.85	-1.20	0.70
	Murder	0.61	-0.06	0.23	-0.38	1.90
	Disputes and assaults	0.05	-0.01	0.93	-1.00	0.94
	Other crimes	0	0		0	0
**Avoidant, dependent, Obsessive-Compulsive Personality Disorder**						
**Gender**	Male	-1.56	0.43	0.03	-18.51	16.43
	female	0	0		0	0
**Education levels**	Elementary	-1.18	0.20	0.05	-2.41	0.67
	Diploma	-0.69	0.20	0.34	-2.56	1.73
	Bachelor's degree and higher	0	0		0	0
**Job status**	Employee	0.31	-0.34	0.66	-9.08	1.85
	Unemployed	0.97	-0.08	0. 05	-0.18	2.01
	Self-employment	0	0		0	0
**Criminal type**	Theft	-0.94	-0.34	0.05	-8.30	0.22
	Financial crime	-1.32	-1.05	0.08	-20.32	0.13
	Murder	-0.80	-1.01	0.26	-18.73	0.56
	Disputes and assaults	-1.67	-1.63	0.05	-20.91	-0.15
	Other crimes	0	0		0	0

## Discussion

These findings are aligned with other studies [10, 14, 15, 21]. Criminal behaviors are very complex and intertwined with political, legal, constabulary, medical, psychological, and social issues [12]. Anti-social characters and psychological traits are constantly associated with criminal and offensive behaviors. Individuals with a high degree of diversity and low cooperation (poor impulse control and lack of empathy) are more likely to have cluster B personality disorder (antisocial, narcissistic, and borderline) [28]. In the study of Wetterborg *et al.*, prisoners with borderline personality disorder have mental health problems, including drug dependence and ADHD (attention deficit hyperactivity disorder), associated with an increased risk of criminal recidivism [29]. A weakness in the control of anger and impulse can lead to stiffness, rape, or physical attack, which is seen in cluster B of personality disorders, leading to severe social and interpersonal problems [2]. In people with cluster B personality disorders, several conditions can lead to anger: intolerance of frustration and intense anger because they are not treated as expected (in narcissistic personality disorder), the need to release tension by using the defense mechanism of splitting, or fear of loss (borderline personality disorder), feeling tired, humiliating others, need for power, lack of empathy, the vital need to attract attention or to be an important member of a group, which can lead to anger (antisocial personality disorder), which subsequently raises the risk of criminal behavior in histrionic personality disorder [2].

In this study, gender was associated with personality disorders; however, the interpretation will not be accurate since the number of women and men were not equal, with fewer women [1]. Regarding the obtained data, the level of education had a significant relationship with clusters A, B, and C personality disorder, especially the level of education in cluster B. According to a study, a multi-item index of health, including physical health, behavioral health, family environment, and psychological health, is significantly related to academic achievement [30]. Impulsivity has a negative relationship with academic success, and impulsivity, emotional instability, and irresponsible lifestyle are observed in cluster B personality disorders, especially borderline disorder [31]. However, according to our knowledge, studies have not been conducted on academic achievement with personality disorders [30].

Unemployment had a significant relationship with cluster C personality disorders. It can be expected that most prisoners are either unemployed or have unstable jobs, and personality disorders are more likely to be related to unemployment, instability and frequent occupation changes, and poorer social quality and occupational performance [32]. Previous studies showed that people with schizotypal and borderline personality disorder have a higher degree of disorders in work, social relationships, and leisure than those with OCPD (Obsessive Compulsive Personality Disorder) and those with moderate avoidant personality disorder [33]. Also, the types of crime, especially theft, was the most frequent among prisoners and had a significant relationship with cluster C. In the study conducted by Fakhrzadegan *et al.*, theft had the highest frequency among the prisoners [16]. Finally, based on the result of the current study, there was a significant relationship between personality disorders (Cluster A, B, and C) and study groups (Table 2). This result is confirmed by other studies [32–34].

## Conclusion

The purpose of this study was to investigate the personality disorders of prisoners. The results of this study showed that there was a significant relationship between the criminal types (disputes and assaults and other crimes) and cluster A (paranoid, schizoid, schizotypal). Furthermore, there was a significant relationship between criminal types (financial crime and theft) and cluster C (avoidant, dependent, obsessive-compulsive personality disorder). Conversely, there was no significant relationship between cluster B (narcissistic, histrionic, antisocial, and borderline personality disorders). Therefore, other studies with bigger samples in different country areas are suggested. 

This present study has several limitations. It is important to consider the nature of any prison population when examining psychiatric issues as most prisoners are young, male, middle class, or unemployed. The small number of women participated in this study should be considered in making any generalization. In addition, the low number of prisoners due to unexpected release and going to other institutions during the study period, lack of information of axis I disorders, mental retardation, and alcohol and drug abuse in the sample, and the cross-sectional nature cannot lead us to any general conclusion.

## Acknowledgments

### Conflict of Interest

The authors declare no conflict of interest.

### Ethical Approval

This study was approved by the Ethics Committee of Kurdistan University of Medical Sciences (IR.MUK.REC.1395/130).

### Consent to participate

Written informed consent was obtained from all participants in the study. 

### Funding

This study was funded by the Research Council of Kurdistan University of Medical Sciences.

### Personal thanks

The author would like to thank all the study participants for completing the questionnaire.

### Authorship:

MAT and FY worked on the design of the proposal. FY contributed to writing the initial draft, analyzing the data, and writing the tables. Also, all authors were involved in reviewing the whole article. All these steps were supervised by MAT.
